# Complete remission of minimal change disease after antiviral monotherapy in a patient with immune-active chronic hepatitis B: A case report

**DOI:** 10.1097/MD.0000000000050999

**Published:** 2026-10-16

**Authors:** Tae Won Lee, Ha Nee Jang, Dae Hyun Song, Sehyun Jung, Seunghye Lee, Se-Ho Chang, Dong Jun Park

**Affiliations:** a Department of Internal Medicine, Gyeongsang National University Changwon Hospital, Changwon, South Korea; b Department of Internal Medicine, Gyeongsang National University College of Medicine, Jinju, South Korea; c Institute of Health Science, Gyeongsang National University, Jinju, South Korea; d Department of Internal Medicine, Gyeongsang National University Hospital, Jinju, South Korea; e Department of Pathology, Gyeongsang National University Changwon Hospital and Gyeongsang National University College of Medicine, Changwon, South Korea.

**Keywords:** antiviral therapy, hepatitis B virus, minimal change disease, nephrotic syndrome, podocytopathy

## Abstract

**Rationale:**

Minimal change disease (MCD) is characterized by diffuse podocyte foot process effacement on electron microscopy, minimal structural changes on light microscopy, and the absence of immune complex deposition on immunofluorescence. In adults, MCD accounts for approximately 10% to 25% of nephrotic syndrome cases and is typically idiopathic, although secondary causes such as infections, medications, malignancies, and immune disorders have been reported. The association between hepatitis B virus (HBV) infection and glomerular disease has been well established in membranous nephropathy and mesangial proliferative glomerulonephritis. However, HBV-associated MCD is rare.

**Patient concerns:**

A 42-year-old woman presented with a 1-week history of generalized edema and weight gain. Physical examination revealed severe bilateral pretibial pitting edema, and laboratory evaluation revealed hypoalbuminemia and nephrotic-range proteinuria.

**Diagnoses:**

The urine protein-to-creatinine ratio was elevated to 4.3, while renal function remained unchanged. Serological testing was positive for hepatitis B surface antigen and hepatitis B e-antigen, with a high viral load (HBV deoxyribonucleic acid 505,437 IU/mL), consistent with immune-active chronic hepatitis B infection. Renal biopsy showed nearly normal glomerular architecture on light microscopy, diffuse podocyte foot process effacement on electron microscopy, and no significant immune complex deposition on immunofluorescence, confirming MCD.

**Interventions:**

Antiviral therapy with tenofovir alafenamide 25 mg orally once daily was initiated and continued throughout the 3-year follow-up period.

**Outcomes:**

Proteinuria progressively improved, and complete remission was achieved at 8 months (urine protein-to-creatinine ratio, 0.2). The patient maintained sustained remission with persistent viral suppression during a 3-year follow-up.

**Lessons:**

Complete remission of MCD was temporally associated with antiviral monotherapy in a patient with immune-active HBV. Although potential confounding factors such as spontaneous remission or the supportive effects of concurrent angiotensin receptor blocker therapy cannot be entirely excluded, this case suggests that viral suppression may play a role in restoring podocyte function. Renal biopsy is essential for accurate diagnosis and treatment planning because corticosteroid therapy may exacerbate viral replication and hepatic injury. Antiviral monotherapy may represent a reasonable initial therapeutic strategy in select patients with biopsy-proven MCD and active hepatitis B infection.

## 1. Introduction

Minimal change disease (MCD) is characterized by diffuse podocyte foot process effacement on electron microscopy, minimal structural changes on light microscopy, and the absence of significant immune complex deposition on immunofluorescence.^[1]^ In adults, MCD accounts for a minority of nephrotic syndrome cases but often presents with sudden generalized edema, hypoalbuminemia, and nephrotic-range proteinuria, frequently necessitating hospitalization.^[2]^ Although most adult MCD cases are idiopathic, secondary forms associated with viral infections, medications, malignancies, and immune-mediated disorders have been documented. The prevalence of MCD in adults is estimated to be 10% to 25%.^[3,4]^

The association between hepatitis B virus (HBV) infection and glomerular disease, particularly membranous nephropathy (MN), mesangial proliferative glomerulonephritis (MPGN), and polyarteritis nodosa, is well documented.^[5]^ In contrast, MCD as a secondary manifestation of HBV infection is extremely rare, and its causal relationship remains unclear. Immune dysregulation and cytokine-mediated inflammatory responses in patients with immune-active HBV infection are hypothesized to induce podocyte dysfunction.^[6]^

A major clinical dilemma arises because corticosteroid therapy, which is the standard treatment for adult MCD, may exacerbate viral replication and worsen hepatitis.^[7]^ Whether antiviral therapy alone can induce MCD remission in patients with high HBV activity remains largely unknown. This case report describes a patient with immune-active chronic HBV infection and biopsy-proven MCD who achieved complete remission (CR) of nephrotic syndrome following antiviral therapy alone.

## 2. Case presentation

A 42-year-old woman presented to the emergency department with a 1-week history of generalized edema and a weight gain of 3 kg. She had no comorbidities other than hypertension and was taking an angiotensin receptor blocker. She denied the use of any other medications, including nonsteroidal anti-inflammatory drugs or herbal supplements. Furthermore, she had no history of recent vaccinations, atopic or allergic diseases, or exposure to other drugs associated with secondary MCD (e.g., lithium or bisphosphonates). There were no symptoms suggestive of hematologic or solid malignancies, such as unexplained fever, night sweats, or significant weight loss, and malignancy screenings performed during follow-up were negative. An extensive diagnostic workup revealed negative results for the other secondary triggers. Specifically, antinuclear antibodies, antineutrophil cytoplasmic antibodies, and phospholipase A2 receptor antibodies antibodies were absent, and complement levels were normal. Screening for human immunodeficiency virus and hepatitis C virus was negative, and paraprotein studies showed no abnormalities. Given the absence of alternative etiologic factors, the clinical focus was directed toward immune-active HBV infection as the most plausible trigger for MCD.

The patient’s family history of kidney disease was unremarkable. The patient denied any recent upper respiratory symptoms, fever, or other signs of infection. Physical examination revealed severe bilateral pretibial pitting edema (grade 4+).

vital signs were as follows: blood pressure, 161/94 mm Hg; heart rate, 75 beats/min; respiratory rate, 18 breaths/min; body temperature, 36.5°C; and oxygen saturation, 100% on room air.

Laboratory evaluation revealed hypoalbuminemia (serum albumin 3.0 g/dL) and hypocalcemia (total calcium 7.9 mg/dL) with preserved renal function (serum creatinine 0.67 mg/dL). Liver enzyme levels were elevated (aspartate aminotransferase 71 U/L; alanine aminotransferase, 89 U/L). Urinalysis revealed persistent 3 + proteinuria without microscopic hematuria. The urine protein-to-creatinine ratio was initially 2.3 g/g and subsequently increased to 4.3 g/g, which was consistent with nephrotic-range proteinuria.

Renal biopsy specimens contained 27 glomeruli for light microscopy and 4 glomeruli for electron microscopy. The exact number of glomeruli evaluated by immunofluorescence microscopy was not available from the original pathology records. The most prominent histological feature was an increase in glomerular size. Although glomerular size was generally enlarged, no global or segmental glomerulosclerosis was observed. Furthermore, the cellularity of the glomerular capillary tufts and the thickness of the capillary walls were within the normal range, and no structural abnormalities, such as fuchsinophilic deposits suggestive of immune complex deposition, spikes in the basement membrane, or tram-track appearances, were observed. Examination of the interstitial and vascular areas revealed no active inflammatory response, such as vascular abnormalities or lymphocytic infiltration. However, focal tubular atrophy was identified, indicating the presence of mild chronic damage within the kidney (Fig. 1). Fluorescence microscopy revealed a faint signal for immunoglobulin M antibodies, but this was judged to be nonspecific (Fig. 2). Electron microscopy revealed no high-density deposits; however, diffuse loss of foot processes was observed (Fig. 3). Serological testing of the patient revealed that both hepatitis B surface antigen and total core antibody were positive. We were unable to perform staining of renal tissue for HBV antigens, which is a limitation of this study.

**Figure 1.**
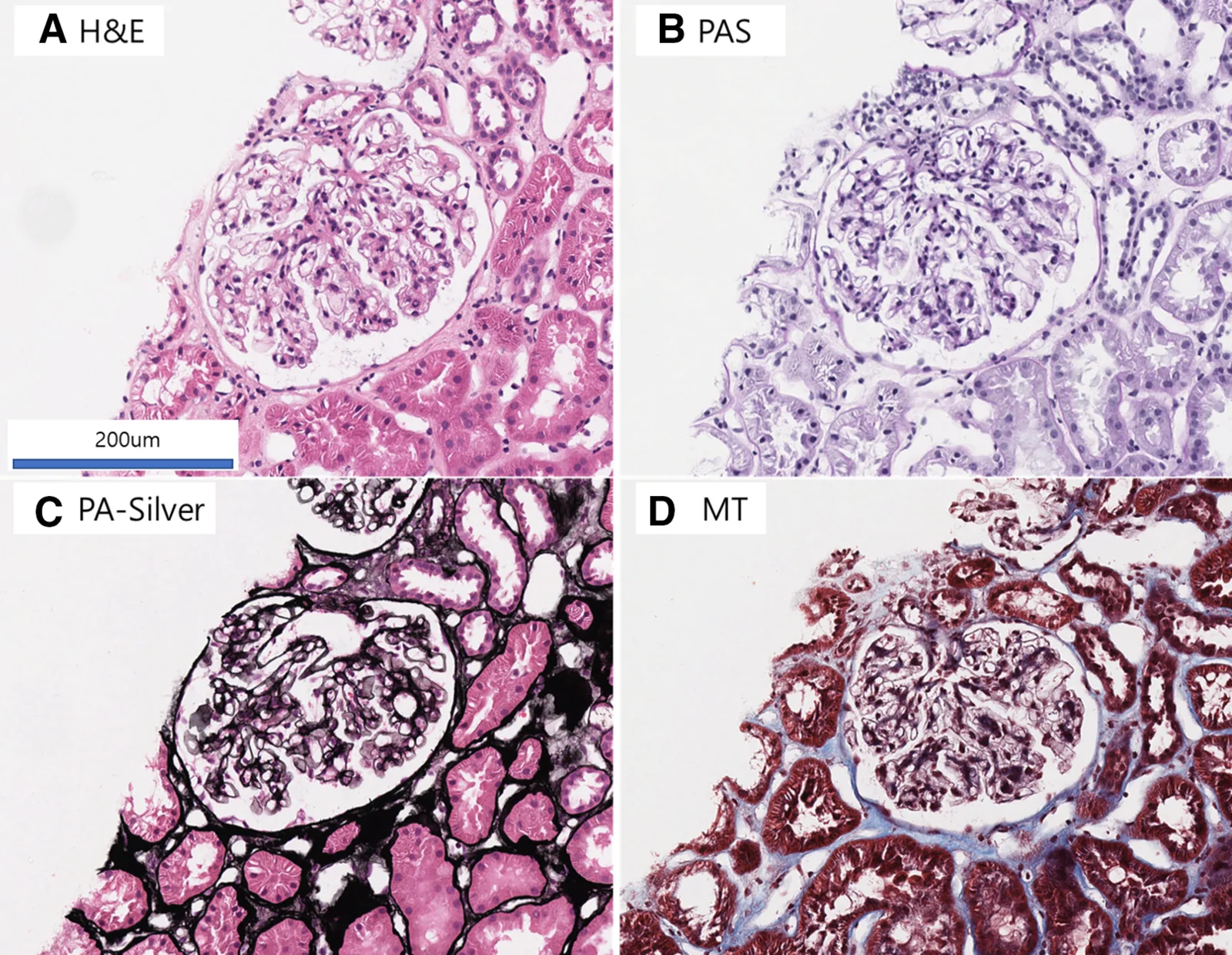
Light microscopy. (A) Hematoxylin and eosin (H&E) stain: Glomerular cellularity was not increased; (B) PAS stain: capillary wall thickness was within normal limits; (C) PA-silver stain: no spikes or tram-track appearances were observed; and (D) Masson-trichrome (MT) stain: there are no fuchsinophilic deposits. Magnification, ×200; scale bars = 200 μm in all panels. PA = periodic acid, PAS = periodic acid–schiff.

**Figure 2.**
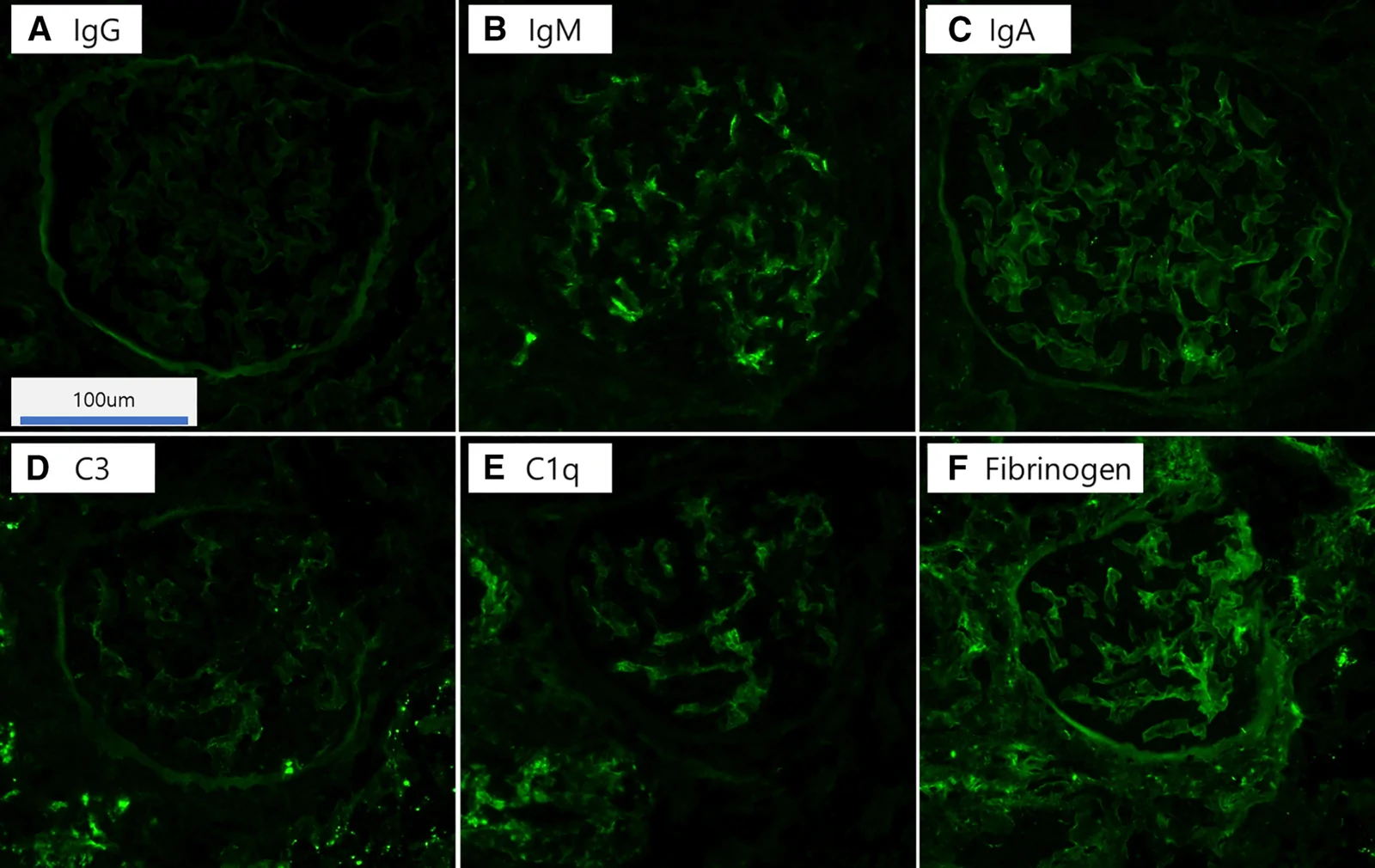
Immunofluorescence microscopy. (A) IgG: there is no significant immunofluorescent deposit; (B) IgM: trace/equivocal staining was observed and was considered nonspecific; (C) IgA: there is no significant immunofluorescent deposit; (D) C3: there is no significant immunofluorescent deposit; (E) C1q: there is no significant immunofluorescent deposit; and (F) fibrinogen: there is no significant immunofluorescent deposit. Magnification, ×200; scale bars = 100 μm in all panels. IgG = immunoglobulin G, IgM = immunoglobulin M.

**Figure 3.**
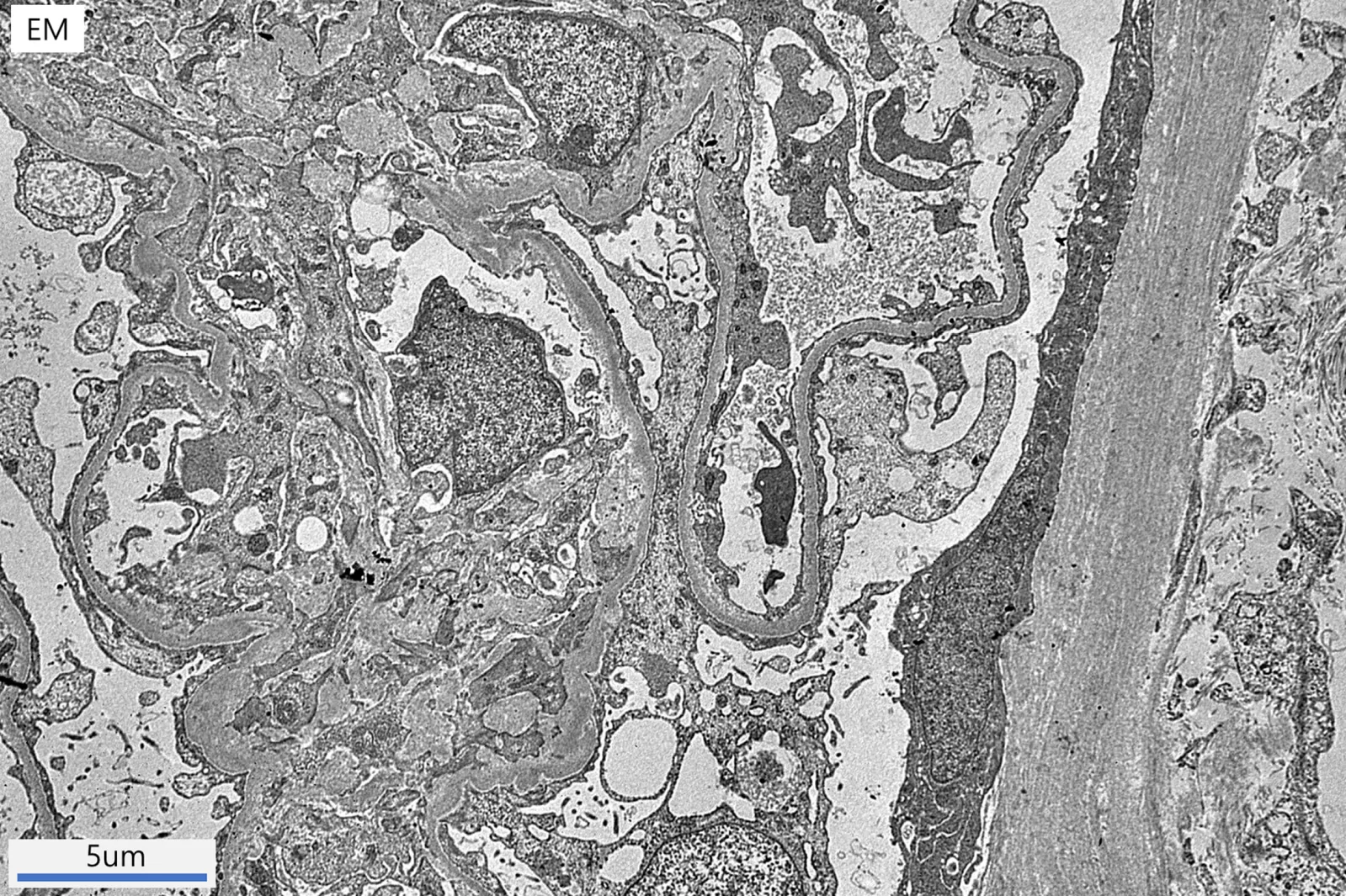
Electron microscopy (EM). No electron-dense deposits and diffuse podocyte foot process effacement. Magnification, ×5000; scale bars = 5 µm.

Serological evaluation revealed hepatitis B surface antigen and hepatitis B e-antigen positivity, anti- hepatitis B e-antigen negativity, and an hepatitis B virus deoxyribonucleic acid (HBV DNA) level of 505,437 IU/mL. Anti-hepatitis B core immunoglobulin M was negative, while anti-hepatitis B core immunoglobulin G was positive, consistent with immune-active chronic hepatitis B. Liver ultrasonography demonstrated a fatty liver, and liver elastography showed no significant fibrosis (5.6 kPa). Based on these findings, antiviral therapy with tenofovir alafenamide (TAF) 25 mg orally once daily was initiated and continued throughout the 3-year follow-up period.

The patient did not receive any corticosteroids or immunosuppressants. Proteinuria gradually decreased, and significant improvement was observed at 10 weeks. At 8 months, urinalysis was negative for protein, and the urine protein-to-creatinine ratio decreased to 0.2 g/g, indicating CR. During follow-up exceeding 3 years, the patient remained in sustained remission, with resolution of proteinuria and marked and sustained virologic suppression (Table 1, Fig. 4).

**Table 1:** Temporal relationship between viral load suppression and proteinuria remission over a 3-year follow-up period.

Timeline/events	Date	Urine protein	UPCR (g/g)	HBV DNA (IU/mL)
Initial presentation				
Admission	2022-06-24	3+	2.3	–
Renal biopsy	2022-06-27	3+	3.2	505,437
Treatment initiation				
Tenofovir started	2022-06-30	3+	3.6	
MCD confirmed	2022-07-22	3+	4.3	
Follow-up				
Posttreatment 6 wk	2022-08-12	3+	4.3	
Posttreatment 10 wk	2022-09-13	1+	0.8	333
Posttreatment 5 mo	2022-12-05	Negative	0.3	272
Posttreatment 8 mo	2023-02-28	Negative	0.2	59
Posttreatment 1 yr	2023-06-13	Negative	0.2	15
Posttreatment 3 yr	2025-06-09	Negative	0.1	10

The lower limit of detection (LLD) for the HBV DNA assay is 10 IU/mL. Values below 10 IU/mL represent target-not-detected (TND). Complete remission (CR) was defined as a UPCR < 0.3 g/g with negative urine dipstick protein.

HBV DNA = hepatitis B virus deoxyribonucleic acid, MCD = minimal change disease, UPCR = urine protein-to-creatinine ratio.

**Figure 4.**
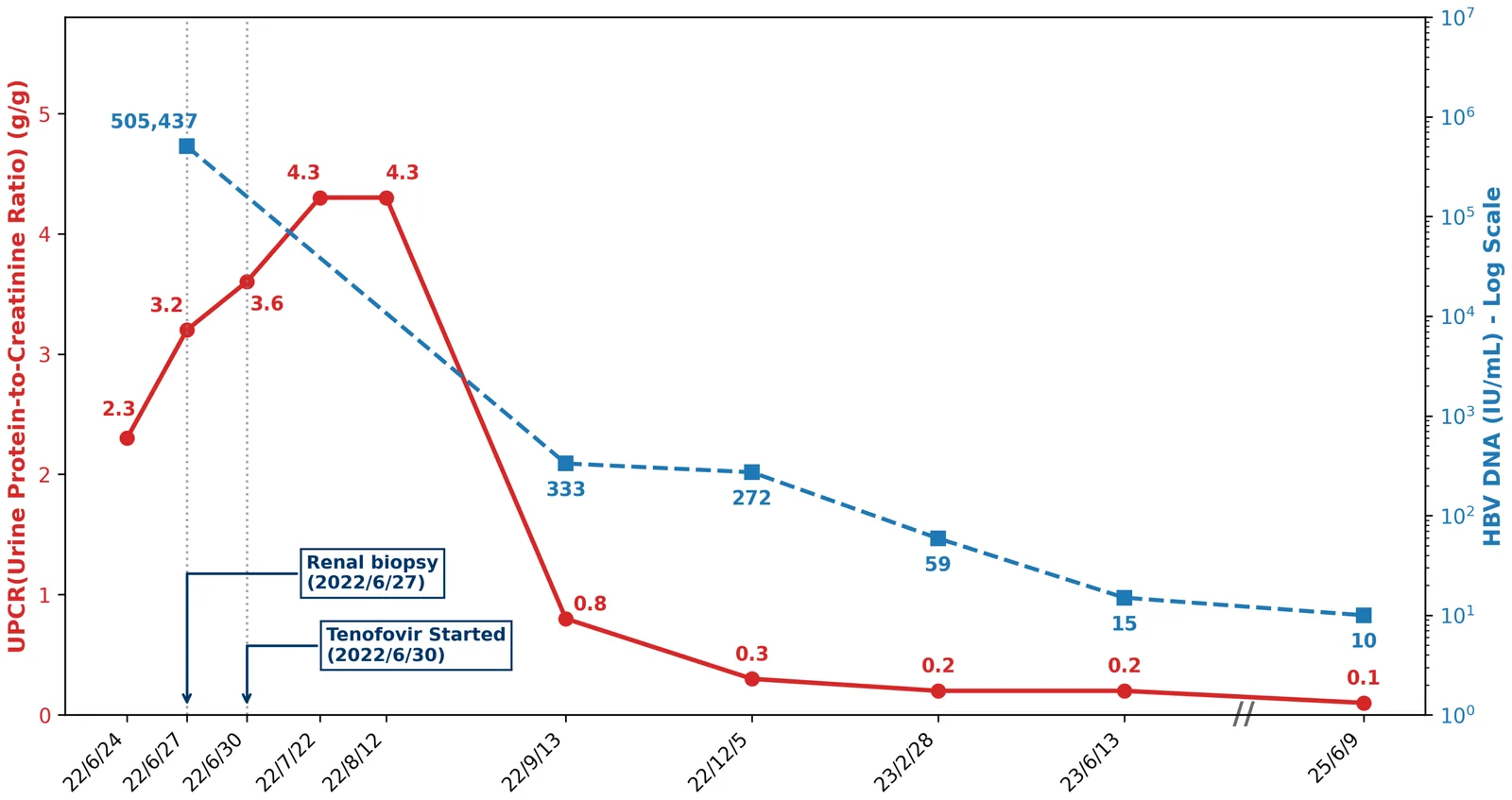
Temporal relationship between viral load suppression and proteinuria remission. HBV DNA = hepatitis B virus deoxyribonucleic acid.

## 3. Discussion

We report a rare case of MCD in a patient with immune-active chronic HBV infection in whom CR of nephrotic-range proteinuria was observed following antiviral monotherapy. This clinical course suggests a potential association between HBV replication and MCD pathogenesis, consistent with the resolution of proteinuria without traditional immunosuppressive agents. This important clinical observation suggested that HBV suppression alone may lead to clinical remission by restoring podocyte function. Previous reports of HBV-associated MCD are scarce and primarily limited to small observational studies or case series. Zhou et al described 4 cases of MCD with HBV infection, suggesting that HBV may be involved in the pathogenesis of podocytopathy. However, their study had several significant limitations, including heterogeneity in treatment strategies, insufficient information on viral activity, and uncertainty in the pathological diagnosis. Therefore, the level of evidence remains limited to determining whether HBV suppression directly contributes to MCD remission.^[8]^

Notably, in a report by Zhou and Jiang, case 4 was treated with a combination of antiviral agents (lamivudine) and systemic steroids (methylprednisolone). Since adult MCD generally responds well to steroids, it is difficult to determine whether the improvement in proteinuria was due to antiviral treatment, the steroid effect, or the combined effect of both therapies. This was a significant confounding factor when interpreting the contribution of the antiviral treatment alone. A notable feature of this case is that MCD remission occurred during TAF monotherapy without concomitant immunosuppressive therapy, thereby reducing 1 important source of treatment-related confounding. TAF offers potent viral suppression and favorable renal safety compared with existing agents, suggesting that it is a favorable treatment option for patients with glomerular disease.^[7,9]^

In the present case, the patient demonstrated excellent treatment adherence and tolerability for clinical management. Adherence was consistently assessed through clinical interviews and pill counts during outpatient visits, and no adverse or unanticipated events related to TAF were observed during the 12-month follow-up period.

The possibility of spontaneous remission in adult MCD patients is an an important consideration. Although spontaneous remission can occur in adult MCD, the temporal association between the initiation of antiviral therapy, progressive HBV DNA suppression, and improvement in proteinuria was noteworthy in this case.^[10,11]^ This temporal association suggests a potential biological link between viral suppression and the recovery of podocyte function. However, because this is a single case description, a causal relationship cannot be definitively proven. The potential contributions of concurrent supportive care, including blood pressure control with an angiotensin receptor blocker, which may exert antiproteinuric effects, as well as the possibility of an unrecognized spontaneous remission, must be acknowledged when interpreting this favorable outcome. Nevertheless, the close chronological proximity between the initiation of antiviral monotherapy, the reduction in viral load, and the resolution of proteinuria remains noteworthy.

The pathophysiological mechanisms by which HBV infection induces podocyte dysfunction remain unclear. However, several potential pathways have been proposed for this process.^[5,12]^ HBV is known to cause T cell dysregulation and an imbalance in cytokine secretion through systemic immune activation. Notably, cytokines, such as interleukin-13, tumor necrosis factor alpha, and interferon gamma, have been implicated in the development of MCD by inducing podocyte cytoskeletal rearrangement and slit diaphragm dysfunction. Furthermore, it has been suggested that viral antigens may directly affect podocytes or induce functional podocytopathy without immune complex deposition, possibly through molecular mimicry. Experimental evidence supports several potential pathways of podocyte injury relevant to MCD. Activated macrophages can induce podocyte injury through tumor necrosis factor alpha-dependent c-Jun amino terminal kinase/p38 signaling, resulting in downregulation of nephrin and podocin.^[13]^ More specifically in the context of HBV infection, HBV X protein has been shown to reduce podocyte adhesion through downregulation of α3β1 integrin.^[14]^ Furthermore, in a rat model, interleukin-13 overexpression induced a minimal-change-like nephropathy characterized by proteinuria and podocyte foot-process fusion, suggesting a potential role for cytokine-mediated podocyte injury.^[15]^

HBV-associated glomerulonephritis has traditionally been considered an immune complex-mediated disease, particularly MN and MPGN.^[16–18]^ This case broadens the pathological spectrum of HBV-associated renal disease to include non–immune complex–mediated functional podocytopathies, such as MCD. Therefore, in patients with HBV infection and nephrotic syndrome, an early renal biopsy should be performed to establish a definitive pathological diagnosis before determining a treatment plan. If MCD is confirmed and the patient is in the immune-active phase, prioritizing antiviral therapy over immunosuppressive therapy may be a reasonable approach. This strategy can also help minimize unnecessary steroid exposure and reduce the associated risk of infection and metabolic complications.

From the patient’s perspective, avoiding corticosteroid therapy provides significant relief. The patient expressed high satisfaction with the treatment outcome, specifically noting recovery from severe edema without weight gain or psychological side effects, which are often associated with long-term steroid use.

We believe that prospective studies are necessary to clarify the true prevalence of MCD in HBV-infected patients and to identify predictors of antiviral treatment response. Although this case provides a clinically informative observation regarding the potential association between viral suppression and MCD remission, it is limited by its single-center design. Thus, further research is required to establish more robust causal relationships. Nevertheless, this clinical observation supports the pathophysiological hypothesis that HBV can act as a trigger or amplifying factor for MCD, and suggests the need to reevaluate treatment strategies for patients with MCD accompanied by HBV infection.

## 4. Conclusion

In this case, a patient with immune-active chronic HBV infection and minimal change disease achieved CR during antiviral therapy alone, without the use of immunosuppressive agents. The temporal association between HBV DNA suppression and improvement in proteinuria suggests a possible relationship between viral activity and podocyte dysfunction; however, causality cannot be established from a single case.

Although HBV-associated renal disease has primarily been recognized as an immune complex–mediated condition (such as MN and MPGN), this case suggests that MCD may represent an additional, non–immune complex–mediated glomerular manifestation associated with HBV infection.

In patients with HBV infection presenting with nephrotic syndrome, renal biopsy is important for establishing the pathological diagnosis and guiding treatment. In selected patients with biopsy-proven MCD and active HBV replication, antiviral therapy without initial immunosuppression may be a reasonable therapeutic approach, although further studies are needed to clarify the relationship between HBV suppression and MCD remission.

## Author contributions

**Conceptualization:** Ha Nee Jang, Sehyun Jung.

**Formal analysis:** Dong Jun Park.

**Investigation:** Ha Nee Jang, Sehyun Jung, Seunghye Lee.

**Methodology:** Sehyun Jung, Dong Jun Park.

**Resources:** Seunghye Lee.

**Supervision:** Tae Won Lee.

**Validation:** Ha Nee Jang.

**Visualization:** Dae Hyun Song.

**Writing – original draft:** Tae Won Lee, Dong Jun Park.

**Writing – review & editing:** Tae Won Lee, Dae Hyun Song, Se-Ho Chang.
